# Walking With a Robotic Exoskeleton Does Not Mimic Natural Gait: A Within-Subjects Study

**DOI:** 10.2196/11023

**Published:** 2019-01-14

**Authors:** Chad Swank, Sharon Wang-Price, Fan Gao, Sattam Almutairi

**Affiliations:** 1 Texas Woman's University Dallas, TX United States; 2 University of Kentucky Lexington, KY United States

**Keywords:** electromyography, gait, kinematics, lower extremity, muscle activation, range of motion, robotic exoskeleton

## Abstract

**Background:**

Robotic exoskeleton devices enable individuals with lower extremity weakness to stand up and walk over ground with full weight-bearing and reciprocal gait. Limited information is available on how a robotic exoskeleton affects gait characteristics.

**Objective:**

The purpose of this study was to examine whether wearing a robotic exoskeleton affects temporospatial parameters, kinematics, and muscle activity during gait.

**Methods:**

The study was completed by 15 healthy adults (mean age 26.2 [SD 8.3] years; 6 males, 9 females). Each participant performed walking under 2 conditions: with and without wearing a robotic exoskeleton (EKSO). A 10-camera motion analysis system synchronized with 6 force plates and a surface electromyography (EMG) system captured temporospatial and kinematic gait parameters and lower extremity muscle activity. For each condition, data for 5 walking trials were collected and included for analysis.

**Results:**

Differences were observed between the 2 conditions in temporospatial gait parameters of speed, stride length, and double-limb support time. When wearing EKSO, hip and ankle range of motion (ROM) were reduced and knee ROM increased during the stance phase. However, during the swing phase, knee and ankle ROM were reduced when wearing the exoskeleton bionic suit. When wearing EKSO, EMG activity decreased bilaterally in the stance phase for all muscle groups of the lower extremities and in the swing phase for the distal muscle groups (tibialis anterior and soleus) as well as the left medial hamstrings.

**Conclusions:**

Wearing EKSO altered temporospatial gait parameters, lower extremity kinematics, and muscle activity during gait in healthy adults. EKSO appears to promote a type of gait that is disparate from normal gait in first-time users. More research is needed to determine the impact on gait training with EKSO in people with gait impairments.

## Introduction

Walking is a complicated process requiring optimal muscle activation and joint mobility to control dynamic balance and posture under different environments. Typified by characteristic muscle activity and kinematic patterns governed by predesigned central nervous system motor programs [1], walking consists of identifiable sequential patterns within a relative timing mechanism [2]. However, an injury to the neuromuscular system is likely to result in atypical walking patterns of both kinematics and muscle activity performance.

Recovery of walking continues to be the primary goal for persons with neurological deficits and a contributing factor to the quality of life [3,4]. Therefore, learning to walk is a major goal during rehabilitation [5,6]. Although the optimal therapeutic intervention to achieve full recovery of gait remains unknown for many patients with neurological injuries, any rehabilitation effort intended to drive neuroplastic changes toward motor recovery should incorporate principles of neuroplasticity. Specifically, inclusion of factors (ie, loading the sole of the foot and attaining adequate hip extension movement) to facilitate appropriate electromyographic (EMG) patterns is thought to be crucial [7]. Locomotor training seeks to capitalize on these established principles [8-10].

Recently, robotic exoskeletons have been developed, and they offer a relatively new form of locomotor training. Robotic exoskeleton devices enable individuals with lower extremity weakness (ie, people with stroke or spinal cord injury) to stand up and walk over ground with a full weight-bearing and reciprocal gait. By adding actuators adjacent to the study participant’s hip and knee joints, robotic exoskeletons provide an external source of controlled joint power. Several exoskeletons have been developed for gait restoration, with much variation in the actuator and sensing technologies. Although there are some commercially available devices, like the ReWalk or EKSO, the technology is not yet mature enough to produce unlimited community ambulation [11-13]. Although gait training with exoskeletons has been shown to be safe and well tolerated, with no significant complications [14] over distances of 40-100 m [15], it is unclear how closely the gait of a person wearing a robotic exoskeleton approximates normal gait. Recently, 2 case studies have highlighted the impact of wearing a robotic exoskeleton on gait characteristics. In the first case study, the lower extremity range of motion (ROM) was generally smaller, with greater hip and knee power generation, for the exoskeleton gait [16]. However, in the second case study, improved symmetry on temporospatial variables and increased gait speed were indicated after robotic exoskeleton gait training in a person with stroke [17].

A common goal of gait retraining is to promote locomotor features typical of normal gait. However, the current robotic exoskeleton devices may promote different nonphysiological walking characteristics. These differences in gait parameters may be accompanied by dissimilarities in kinematics and muscle activity typically observed in normal walking. It is crucial to identify the differences between exoskeleton walking and normal walking prior to using a robotic exoskeleton system for gait training. Therefore, the purpose of this study was to examine whether wearing a robotic exoskeleton suit affects kinematics and muscle activity of the lower extremities during walking. We compared healthy individuals’ gait parameters under 2 conditions: normal walking and walking while wearing a robotic exoskeleton suit.

## Methods

### Participants

Healthy adults 18-70 years old without any neurological disorder were recruited from the local community. Exclusion criteria was based, in part, on the limitations of the robotic exoskeleton, EKSO (Ekso Bionics, Richmond, CA, USA) used in this study and included: (1) screening failure of EKSO frame limitations (weight ≤100 kg; 1.58-1.88 m tall; standing hip width ≤41.9 cm; near-normal ROM in hips, knees, and ankles; and leg length discrepancy ≤1.9 cm), (2) severe spinal instability, (3) unresolved deep vein thrombosis, (4) orthostatic hypotension, (5) skin integrity issues on contact surfaces of the device or sitting surfaces, (6) significant cognitive impairments (unable to follow 3-step commands), and (7) pregnancy.

### Instrumentation

**Figure 1:**
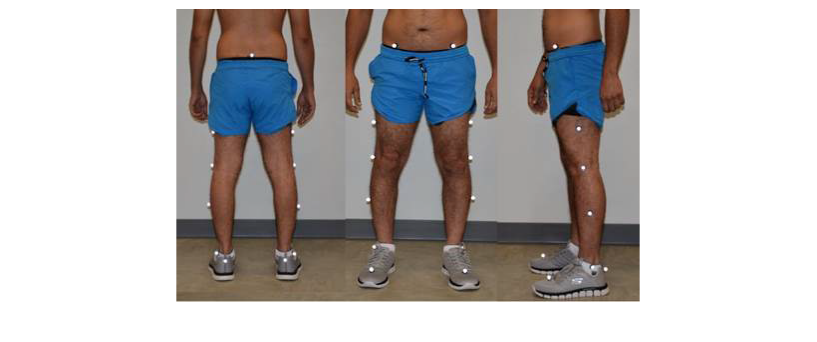
VICON Plug-In-Gait model lower extremity marker placements.

A 10-camera VICON Motion Analysis System (Vicon Motion Systems Inc, Centennial, CO, USA) was used to capture kinematic data. The sampling rate of the 10 cameras was set at 120 Hz, and the cameras were time-synchronized with 6 AMTI (Waterton, MA, USA) force plates, for which the sampling rate was set at 1200 Hz. The force plates were placed in the middle of the 9-m walkway. The threshold of the force plates was set at 10 N in order to determine gait events (ie, heel strikes and toe offs). A VICON Plug-In-Gait model with 15 reflective markers was used to obtain joint motions of lower extremities. Marker placements are shown in Figure 1.

EMG data were obtained from the right and left gluteus medius, rectus femoris, medial hamstrings, tibialis anterior, and soleus muscles with 10 wireless surface electrode pairs (Delsys Trigno EMG system (Delsys Inc, Natick, MA, USA)). The bandwidth of the EMG system was set at 20-450 Hz with a gain of 1000. The Delsys Trigno EMG system contains a notch filter to eliminate nonphysiological signals. The EMG signal was recorded at a sampling rate of 960 Hz and was time-synchronized with the VICON Motion Analysis System. The 10 surface electrode pairs were affixed with self-adhesive tape on the specific location for each muscle following the Surface ElectroMyoGraphy for the Non-Invasive Assessment of Muscles [18] recommendation to minimize surface myoelectric signal cross-talk [19]. Prior to electrode placement, the patient’s skin in the areas of electrode placement was cleaned with isopropyl alcohol. If there were excessive hair, a new disposable razor was used to shave the hair to improve the quality of the EMG recording.

### Procedures

After the participants signed a written consent form approved by the Institutional Review Board of Texas Woman’s University, they completed an intake form for their demographic data (age, gender, and leg dominance); past medical history; past surgical history; and activity level. The investigator then took anthropometric measurements of each participant. These measurements, including height; weight; standing hip width; ROM in hips, knees, and ankles; and leg length, were used to ensure that the participants were able to fit the exoskeleton suit, EKSO. Other anthropometric measurements, including leg length, knee width, and ankle width, were taken as required for the VICON Plug-In-Gait model.

Preceding walking trials for each condition (with and without wearing EKSO), a static trial was captured to create a customized lower-body model for each participant based on the VICON Plug-In-Gait model. For walking trials, the participants were asked to wear a pair of shorts and a pair of tennis shoes, required for EKSO. Kinematic and EMG data were collected simultaneously. During each walking trial, each participant was asked to look straight ahead, if possible, and to walk at a self-selected speed on a 9-m level walkway. Participants stepped onto the force plates on their 4^th^ or 5^th^ steps after attaining a constant velocity. For each of the 2 conditions (with and without wearing the EKSO), data from 5 walking trials were collected from each participant. Prior to trials with EKSO, each participant was given instructions and allowed to practice walking with an EKSO-trained therapist for a minimum of 15 minutes and until the therapist was comfortable providing only close supervision to prevent loss of balance.

### Signal Processing

First, the collected data were processed using the VICON Nexus software to label markers; interpolate, as necessary, for missing data points; determine the gait events; and, finally, generate C3D files. Each walking trial was divided into individual gait cycles that began and ended with the heel strike of the same foot, and then the data were normalized in time by the percent of the gait cycle. Each complete gait cycle was further divided into a stance phase (%) and a swing phase (%). Next, customized MATLAB (Mathworks Inc, Natick, MA, USA) scripts were used to process the C3D files and to generate sagittal joint angles of the hip, knee, and ankle as well as temporospatial variables.

Similarly, custom MATLAB scripts were used to process and produce surface EMG (sEMG) amplitudes for each walking trial. Root mean square (RMS) values of EMG were used to quantify the amount of EMG activity for each walking trial. EMG RMS values were obtained using a window size of 120 samples with 60 samples overlapping. Then, sEMG RMS values were normalized across a complete gait cycle (100%) with 101 data points over the corresponding phase. Finally, sEMG RMS values were further normalized with respect to the peak over the gait cycle. The peak EMG value of the corresponding stance or swing phase of each walking trial was used for the normalization of EMG values. We elected to use the peak EMG normalization approach in order to allow for comparison with previous studies and replication with different neurologically impaired patient populations (ie, stroke and spinal cord injury) [20].

During walking, joint motion predominantly occurs in the sagittal plane. In particular, when using an exoskeleton, motions in other planes are further restrained (reduced). Thus, we are primarily interested in the motion of the sagittal plane. It should be noted, however, that muscles typically cross joints with actions not strictly limited to certain anatomical planes. Hence, muscles routinely cause motion in all 3 planes. For example, frontal plane stability is critical in the single-leg support phase of walking. Therefore, we elected to look at muscle groups characteristically involved in walking regardless of their primary plane of action.

### Statistical Analysis

All statistical analyses were performed using SPSS version 24 (IBM Corp, Armonk, NY, USA). Descriptive statistics were performed to describe participants’ demographic data and gait parameters. Average values of all of the complete gait cycles and 5 walking trials were included in statistical analysis to minimize individual trial variations. Temporospatial parameters of gait were analyzed using paired *t* tests. With regard to kinematic data, because there were no significant differences between the left and right lower extremities, the averages of the right and left maximal sagittal ROM of the hip, knee, and ankle joints were used for statistical analysis. Therefore, 2 separate 2 (condition) ×3 (ROM) repeated measures analyses of variance (ANOVAs) were used to analyze kinematic variables: 1 for the stance and 1 for the swing phase. Due to significant left and right lower extremity differences in EMG, each limb was analyzed separately for stance and swing phases. Therefore, 4 separate 2 (condition) ×5 (muscle) repeated measures ANOVAs were used to analyze the EMG data: 2 for the stance and 2 for the swing phase, respectively. The alpha level was set at .05 for all statistical analyses.

An *a priori* power analysis using G*Power [21], with considerations of the use of *F* test, within factor design, and an anticipated large effect size (.4), indicated the necessary sample size was 15 participants to achieve a power of .80.

## Results

The study was completed by 15 participants, 6 males and 9 females, with an average age of 26.2(SD 8.3) years (range, 19-50 years), average height of 171.8 (SD 7.9) cm (range, 161-184.5 cm), and average weight of 65.8 (SD 11.4) kg (range, 54.5-93.5 kg). Right leg dominance was reported by 12 patients. Participants demonstrated significant differences (*P*<.001) between conditions (with and without EKSO) on all temporospatial gait parameters (Table 1). Overall, participants wearing EKSO walked slower, with shorter steps and greater double-limb support time.

**Figure 2:**
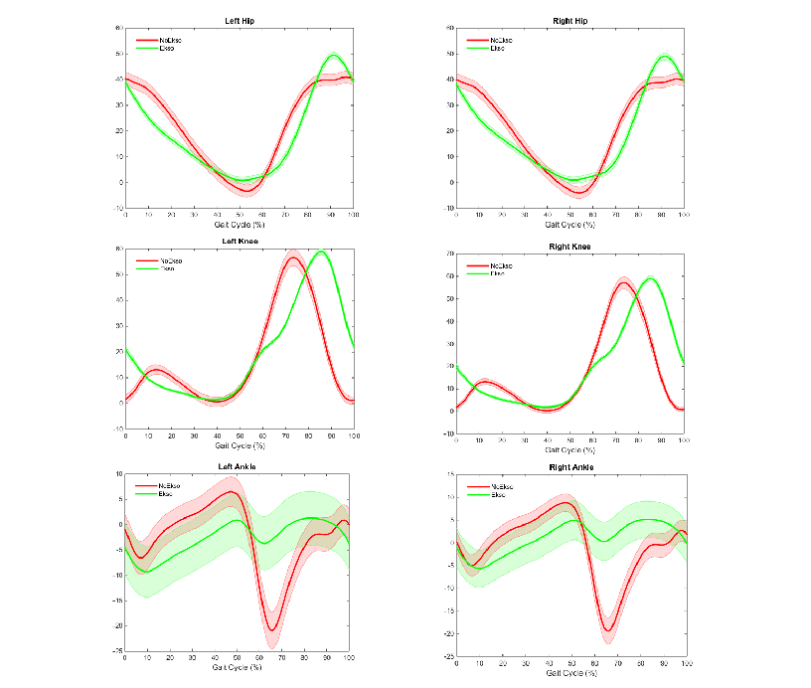
Mean (SE) lower extremity joint motion across the gait cycle. y-axis: range of motion..

Table 2 lists the maximal sagittal ROM at the hip, knee, and ankle joints for the stance and the swing phases. ANOVA showed differences between with and without EKSO conditions. In the stance phase, there were significantly less hip and ankle motions but greater knee motions on both lower extremities for the EKSO condition. ANOVA results also revealed significant differences between the 2 conditions in the knee and ankle motions in the swing phase but not in the hip motion. Specifically, walking with EKSO produced equivalent hip motions but less knee and ankle motions bilaterally in the swing phases. Figure 2 demonstrates lower extremity joint motion across the gait cycle.

**Figure 3:**
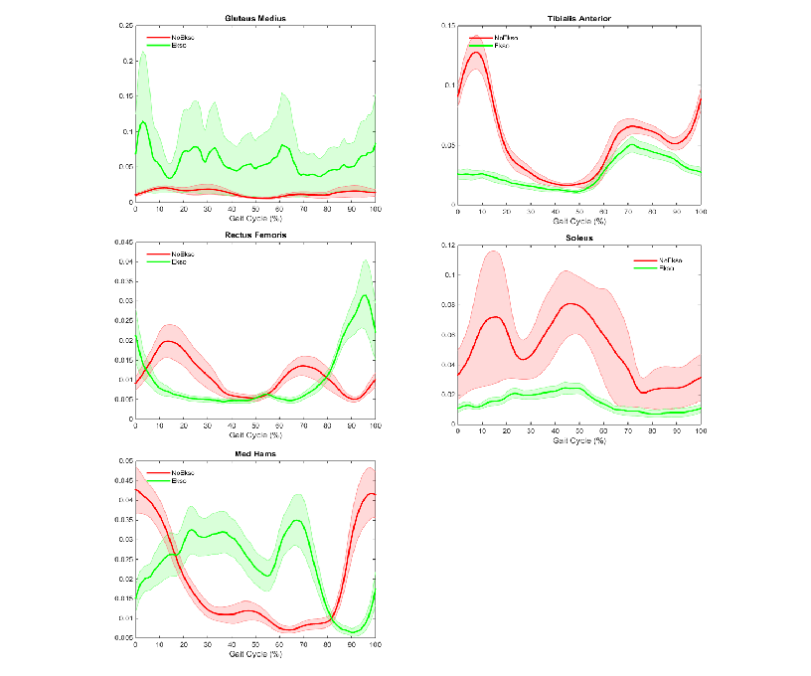
Mean (SE) right lower extremity electromyographic muscle activity across the gait cycle. med hams: medial hamstrings; y-axis: volt.

**Figure 4:**
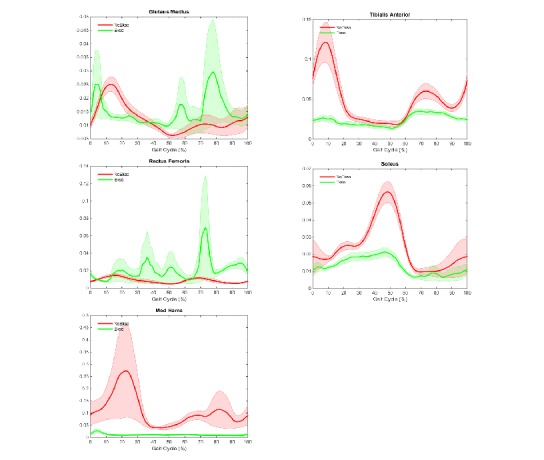
Mean (SE) left lower extremity electromyographic muscle activity across the gait cycle. med hams: medial hamstrings; y-axis: volt.

Table 3 lists sEMG RMS values (%) of the 10 muscles for the stance and the swing phases. In the stance phase, ANOVA results showed significant differences between with and without EKSO conditions for all lower extremity muscle groups bilaterally. In the swing phase, ANOVA results showed significant differences between with and without EKSO conditions only for the distal muscle groups (bilateral soleus and tibialis anterior) and left medial hamstrings. Figures 3 and 4 show lower extremity muscle activity across the gait cycle.

**Table 1:** Temporospatial gait parameters.

Parameter	Without EKSO^a^, mean (SD)	With EKSO, mean (SD)	*P* value
Speed (m/s)	1.32 (0.16)	0.31 (0.04)	<.001
Stride length (m)	1.41 (0.12)	0.72 (0.14)	<.001
Double-limb support (s)	0.17 (0.02)	0.45 (0.06)	<.001
Left step length (m)	0.69 (0.06)	0.34 (0.01)	<.001
Right step length (m)	0.72 (0.06)	0.31 (0.16)	<.001

^a^Robotic exoskeleton

**Table 2:** Sagittal range of motion of lower extremity during gait with and without wearing a robotic exoskeleton (EKSO).

Lower extremity	Stance phase			Swing phase		
	Without EKSO, mean (SD)	With EKSO, mean (SD)	*P* value	Without EKSO, mean (SD)	With EKSO, mean (SD)	*P* value
Hip	44.33 (5.11)	37.90 (3.39)	<.001^a^	42.42 (4.92)	43.08 (4.55)	0.69
Knee	23.07 (4.52)	28.62 (5.39)	.006^a^	56.89 (8.24)	40.68 (4.07)	<.001^a^
Ankle	18.39 (2.44)	11.74 (2.21)	<.001^a^	24.07 (7.13)	6.85 (2.16)	<.001^a^

^a^Significant at *P*<.05.

**Table 3:** Amplitude of lower extremity electromyographic muscle activity during gait with and without wearing a robotic exoskeleton.

Muscle	Stance phase					Swing phase						
	Without EKSO, mean (SD)	With EKSO, mean (SD)	*P* value	Difference, %		Without EKSO, mean (SD)		With EKSO, mean (SD)		*P* value		Difference, %
Right gluteus medius	0.62 (0.07)	0.55 (0.05)	.003^a^		−11.3	0.59 (0.13)	0.62 (0.10)		.47		6.9	
Right rectus femoris	0.66 (0.08)	0.54 (0.07)	.001^a^		−18.2	0.59 (0.08)	0.56 (0.11)		.42		−5.1	
Right medial hamstring	0.64 (0.070)	0.55 (0.09)	.01^a^		−15.6	0.42 (0.06)	0.41 (0.09)		.62		−2.4	
Right tibialis anterior	0.61 (0.04)	0.49 (0.06)	<.001^a^		−19.7	0.59 (0.11)	0.68 (0.11)		.02^a^		15.3	
Right soleus	0.61 (0.06)	0.54 (0.09)	.003^a^		−11.5	0.43 (0.15)	0.63 (0.13)		.004^a^		46.5	
Left gluteus medius	0.63 (0.04)	0.560 (0.05)	.001^a^		−11.1	0.61 (0.08)	0.64 (0.07)		.22		4.9	
Left rectus femoris	0.64 (0.06)	0.54 (0.09)	.003^a^		−14.3	0.57 (0.10)	0.55 (0.13)		.72		−1.8	
Left medial hamstring	0.62 (0.09)	0.52 (0.09)	.007^a^		−16.1	0.59 (0.09)	0.52 (0.10)		.04^a^		−13.6	
Left tibialis anterior	0.62 (0.08)	0.56 (0.10)	.03^a^		−9.7	0.60 (0.08)	0.71 (0.06)		<.001^a^		18.3	
Left soleus	0.60 (0.04)	0.52 (0.07)	.004^a^		−13.3	0.42 (0.14)	0.62 (0.13)		0.01^a^		47.6	

^a^significant at <.05

## Discussion

### Principal Findings

The results showed that walking with EKSO was dissimilar to typical walking with regard to lower extremity muscle activity and joint motions as well as temporospatial gait parameters. Overall, the participants in this study walked with EKSO at approximately one-fourth their average walking speed and with nearly half the stride length. These changes likely contributed to an increase in double-limb support time. Although the participants were instructed to walk at a self-selected pace during each condition, the participants were unable to match their typical walking performance when walking with EKSO. A possible explanation for these observed differences is the lack of training of our participants for walking with EKSO. Even though the participants were instructed on how to initiate a step and given 15 minutes of practice time, this short training may not have been sufficient to allow the participants to reach optimal exoskeleton gait performance. Moreover, a second possible explanation for the differences observed in temporospatial parameters is the technological limitations of the current robotic exoskeletons. Specifically, mechanical design and actuators of contemporary exoskeletons are known to limit gait performance and capacity [22]. For instance, actuators are located at the hip and knee joints but not at the ankle joints. Contemporary ankle joints are typically either a fixed solid plate or tension spring motion plates.

With changes in temporospatial parameters, it was expected that muscle activity would be impacted as well. It has been shown that walking at slower speeds resulted in decreased muscle activity of the lower extremities during both the stance and swing phases of gait regardless of age [23]. Similarly, we observed an average reduction of nearly 15% in muscle activity during the stance phase in both lower extremities (Table 3). This reduction may have been caused by the reduction in speed or by the structural support provided by the EKSO device. However, we did not see a similar reduction in muscle activity in the swing phase. On the contrary, an increase of 32% in muscle activity of the distal lower extremity was observed in our study. We speculate that the increase in muscle activity in our study was a result of EKSO’s mechanical constraints [22]. In particular, the EKSO footplate limits ankle motion, and this may have required participants to compensate for the reduced ankle mobility.

Beyond limiting ankle ROM, we observed several changes in lower extremity kinematics when walking with EKSO as compared to when walking without EKSO. During the stance phase, we observed less hip and ankle motions but greater knee motion when wearing EKSO. In the swing phase, we observed less knee and ankle motions, but no difference in hip motion when wearing EKSO. Overall, it appears that gait with EKSO produced a pattern where shorter steps due to limited ankle motion contributed to a shortened trailing limb. While a typical swing phase ankle arc of motion moves from maximal plantar flexion in the initial swing to a near ankle neutral position in the terminal swing, EKSO-induced shortened steps minimized the potential for ankle plantar flexion in order to accommodate a relatively fixed footplate. Moreover, the limited ankle motion also likely required greater knee flexion during the stance phase. The EKSO footplate and corresponding upright support do not allow for optimal ankle joint motion. Rather, the mechanical constraints of EKSO ankle joint appear to influence lower extremity kinematics as well as corresponding muscle activity.

Our participants were without injury and, when not wearing EKSO, demonstrated walking parameters consistent with typical gait. For individuals with neurological dysfunction, return to walking is the primary focus of rehabilitation. As previously reported, gait training after neurological injury should include proper loading of the sole of the foot and attaining adequate hip extension to facilitate appropriate muscle activation [7]. The findings of this study question whether mechanical constraints in the current versions of robotic exoskeletons preclude the possibility of promoting kinematics suitable to induce satisfactory muscle activity. Our participants were novice EKSO users who were tested during their first session of wearing EKSO. It is possible that a longer training time with EKSO might have promoted more typical EMG patterns despite the mechanical constraints. Additionally, people with biomechanical limitations from various neurological and orthopedic injuries are able to walk albeit with an altered gait cycle and atypical muscle activity [24-26]. Although EKSO does not appear to promote normal gait, it may stimulate an altered functional gait. Although an altered gait is potentially less efficient [27], this functional gait may meet the mobility objectives of a person recovering from a neurological injury.

### Limitations

There are several limitations in this study. First, our sample of 15 participants was primarily young, active individuals, and this may have limited generalizability of our conclusions. Second, the preferred EMG normalization method is to use a single maximum muscle test for each muscle group tested. We utilized a peak EMG normalization approach as this may be particularly appropriate when patient populations most likely to use a robotic exoskeleton (ie, spinal cord injury) are being studied because maximal muscle testing may be prohibitive [20]. Third, the intended user of EKSO is an individual with locomotion disabilities. Although outside the scope of this study, examination of gait parameters of individuals with locomotion disabilities is recommended for future studies. Further, EKSO requires the use of an assistive device (cane, walker, or forearm crutches) while walking. In this study with healthy individuals, we elected not to use an assistive device but provided close supervision to prevent a loss of balance. The use of an assistive device, as recommended by robotic exoskeleton companies, may have further altered gait kinematics and muscle activity. Lastly, walking speed was not controlled in this study. Future studies should consider exploring gait parameters and related asymmetries under normal walking and EKSO walking conditions while controlling speed.

### Conclusion

EKSO appears to promote a type of gait that is disparate from normal gait in first-time users. Specifically, the mechanical constraints of EKSO appeared to alter joint motion and influence muscle activity throughout the gait cycle. These changes resulted in a walking pattern characterized by slower speeds, smaller steps, and less single-limb stance time. Given this foundation, more research will be necessary to determine the impact of wearing a robotic exoskeleton on rehabilitation in people with gait impairment.

## Abbreviations

EMG: electromyography
RMS: root mean square
ROM: range of motion
sEMG: surface electromyography
